# Nafamostat has anti-asthmatic effects associated with suppressed pro-inflammatory gene expression, eosinophil infiltration and airway hyperreactivity

**DOI:** 10.3389/fimmu.2023.1136780

**Published:** 2023-04-21

**Authors:** Venkata Sita Rama Raju Allam, Ida Waern, Sowsan Taha, Srinivas Akula, Sara Wernersson, Gunnar Pejler

**Affiliations:** ^1^ Department of Medical Biochemistry and Microbiology, Uppsala University, Uppsala, Sweden; ^2^ Department of Anatomy, Physiology and Biochemistry, Swedish University of Agricultural Sciences, Uppsala, Sweden

**Keywords:** nafamostat, serine proteases, asthma, house dust mite, cytokines, airway hyperreactivity, inflammation

## Abstract

**Introduction:**

Asthma is characterized by an imbalance between proteases and their inhibitors. Hence, an attractive therapeutic option could be to interfere with asthma-associated proteases. Here we exploited this option by assessing the impact of nafamostat, a serine protease inhibitor known to neutralize mast cell tryptase.

**Methods:**

Nafamostat was administered in a mouse model for asthma based on sensitization by house dust mite (HDM) extract, followed by the assessment of effects on airway hyperreactivity, inflammatory parameters and gene expression.

**Results:**

We show that nafamostat efficiently suppressed the airway hyperreactivity in HDM-sensitized mice. This was accompanied by reduced infiltration of eosinophils and lymphocytes to the airways, and by lower levels of pro-inflammatory compounds within the airway lumen. Further, nafamostat had a dampening impact on goblet cell hyperplasia and smooth muscle layer thickening in the lungs of HDM-sensitized animals. To obtain deeper insight into the underlying mechanisms, a transcriptomic analysis was conducted. This revealed, as expected, that the HDM sensitization caused an upregulated expression of numerous pro-inflammatory genes. Further, the transcriptomic analysis showed that nafamostat suppressed the levels of multiple pro-inflammatory genes, with a particular impact on genes related to asthma.

**Discussion:**

Taken together, this study provides extensive insight into the ameliorating effect of nafamostat on experimental asthma, and our findings can thereby provide a basis for the further evaluation of nafamostat as a potential therapeutic agent in human asthma.

## Introduction

Asthma is a non-communicable, heterogeneous chronic airway inflammatory disorder affecting ~300 million people worldwide. Asthma is characterized by airway inflammation and airway remodelling associated with various symptoms, including shortness of breath, wheezing, cough and chest tightness, and variable expiratory airflow limitation (1–4). The progression of asthma is complicated and is associated with a diverse interplay between environmental factors and genetic predispositions, thereby stratifying asthmatics into distinct phenotypes with significant clinical variance (1). Further, asthma endotyping is an emerging concept, through identifying different cellular and molecular mechanisms involved in the disease pathogenesis (2). Current therapies for asthma include a combination of anti-inflammatory agents like corticosteroids along with bronchodilators; however, the poor response to these regimens among certain asthmatic subsets and the adverse side-effects associated with their long-term use emphasizes the need to identify novel therapeutic interventions to treat asthma effectively (4).

Asthma is a multifaceted disease primarily allied with an altered Th2/Th17 immune system in response to environmental allergens (5). Proteolytic events mediated by a variety of proteases represent one of the mechanisms involved in the pathogenesis of asthma, having an impact at multiple levels, including production of inflammatory mediators, induction of airway leukocyte infiltration, airway hyperreactivity (AHR) and airway remodelling (6, 7). Such proteases can be derived either from exogenous sources such as environmental allergens, or can be produced endogenously by various immune cells. For example, mite allergens include several serine proteases such as Dermatophagoides *pteronyssinus* 3 (Der p 3), Der p 6 and Der p 9, and the cystine proteases Der p 1 and Der farina (8, 9). These proteases have the capacity to proteolytically disrupt tight junctions of the epithelial cell barrier, thereby increasing epithelial permeability and facilitating the access of antigen-presenting cells to antigen (10, 11). This can stimulate and activate various downstream inflammatory signalling pathways resulting in the induction of an immune response to the allergen.

Similar to the pathogenic role of the exogenous proteases, endogenous proteases released from various immune cells in response to allergen stimuli can also play a prominent role in the initiation and progression of allergic airway inflammation. Such endogenous proteases include chymases, tryptases and carboxypeptidase A3 from mast cells, elastase, cathepsin G and myeloblastin (proteinase 3) from neutrophils, bronchial epithelial cell-derived transmembrane protease serine 11D (TM11D; human airway trypsin-like protease), granzymes expressed by cytotoxic T lymphocytes and NK cells, as well as matrix metalloproteases expressed by epithelial and other inflammatory cells (6, 12–16). Such proteases can modulate allergic airway inflammation by affecting various targets, such as pattern recognition receptors (e.g., TLR4), protease-activated receptors (PARs) including PAR2, various extracellular matrix components and inflammatory mediators. This can promote various processes, including mast cell degranulation, inflammatory cell recruitment, release of AHR-promoting bronchoconstrictors and proliferation of fibroblasts and smooth muscle cells, the latter contributing to airway thickening and airway remodelling (6, 15, 17).

To mitigate the detrimental effects attributed to both exogenous and endogenous proteases, the human body is equipped with a range of endogenous protease inhibitors (EPIs). EPIs such as cystatin A and SPINK5 maintain the epithelial barrier integrity, thereby blocking inflammatory pathways and reducing the risk of allergic sensitization (18–20). Earlier findings have reported that there may be an imbalance between the corresponding proteases and EPIs in various pathological conditions. Hence, deficiency of certain EPIs has been observed in certain asthma endotypes due to genetic predisposition, resulting in exacerbated inflammation (19, 21). To compensate for such a protease-antiprotease imbalance, various synthetic protease inhibitors have been assessed for their ability to intervene with inflammatory conditions such as asthma (21, 22).

Nafamostat mesylate is such a synthetic inhibitor, with the ability to target multiple types of serine proteases, but with selectivity for mast cell tryptase (23). Nafamostat is in clinical use for the treatment of pancreatitis, but has also been considered as a potential therapeutic in other types of inflammatory settings (24–26). Earlier studies using various experimental models of allergic asthma in mice demonstrated an anti-asthmatic effect of nafamostat (27–29). However, the exact molecular and cellular consequences of nafamostat treatment in the attenuation of asthma remain poorly characterized and there is thus a need for a further understanding of this issue. In the present study, we therefore performed a detailed investigation of the therapeutic potential of nafamostat in a house dust mite (HDM)-based mouse model for asthma. Our findings reveal that nafamostat alleviates multiple hallmark features of asthma, including the suppression of gene expression patterns associated with inflammation.

## Results

### Nafamostat abrogates HDM-induced airway inflammation and airway hyperreactivity

**Figure 1:**
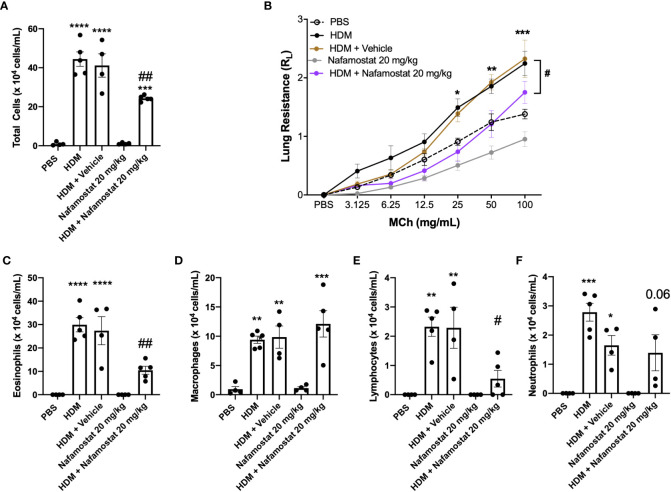
Nafamostat inhibits airway inflammation and AHR in HDM-induced experimental asthma. Mice received either PBS or HDM twice a week for 6 weeks. Mice were treated with vehicle or with nafamostat (20 mg/kg) 30 min prior to each HDM extract instillation. Control mice were treated with PBS only. Total cells **(A)**, eosinophils **(C)**, macrophages **(D)**, lymphocytes **(E)** and neutrophils **(F)** were measured in the BAL fluid. Lung resistance (R_L_) **(B)** was measured using a Buxco FinePointe series instrument. Data represent mean values ± SEM. *P < 0.05, **P < 0.01, ***P < 0.001 and ****P < 0.0001 vs. the PBS group. ^#^ *P* < 0.05 and ^##^ *P* <.01 vs. the HDM group. n = 4 – 5 mice per group. HDM, house dust mite.

To study the impact of nafamostat in allergic airway inflammation, we used a protocol based on sensitization with house dust mite (HDM) extract. The used model was shown to replicate major features of the clinical asthma, including an increased cell infiltration to the airway lumen (**Figure 1A**) and raised airway hyperreactivity (AHR) in response to methacholine (**Figure 1B**). In contrast, no effects of HDM sensitization on dynamic compliance (Cdyn) was seen (**Supplementary Figure 1**). The increase in inflammatory cell infiltration to the airway lumen was primarily due to a profound increase of eosinophils (**Figure 1C**) but increased infiltration was also seen for macrophages, lymphocytes and neutrophils (**Figures 1D–F**) as compared to control mice. Treatment of HDM-sensitized mice with nafamostat significantly reduced the airway inflammation and lung resistance (R_L_) (**Figures 1A, B**), which was accompanied by a substantial reduction in the infiltration of eosinophils (**Figure 1C**) and lymphocytes (**Figure 1E**) to the airway lumen, whereas a tendency of reduced neutrophil influx was seen (**Figure 1F**).

**Figure 2:**
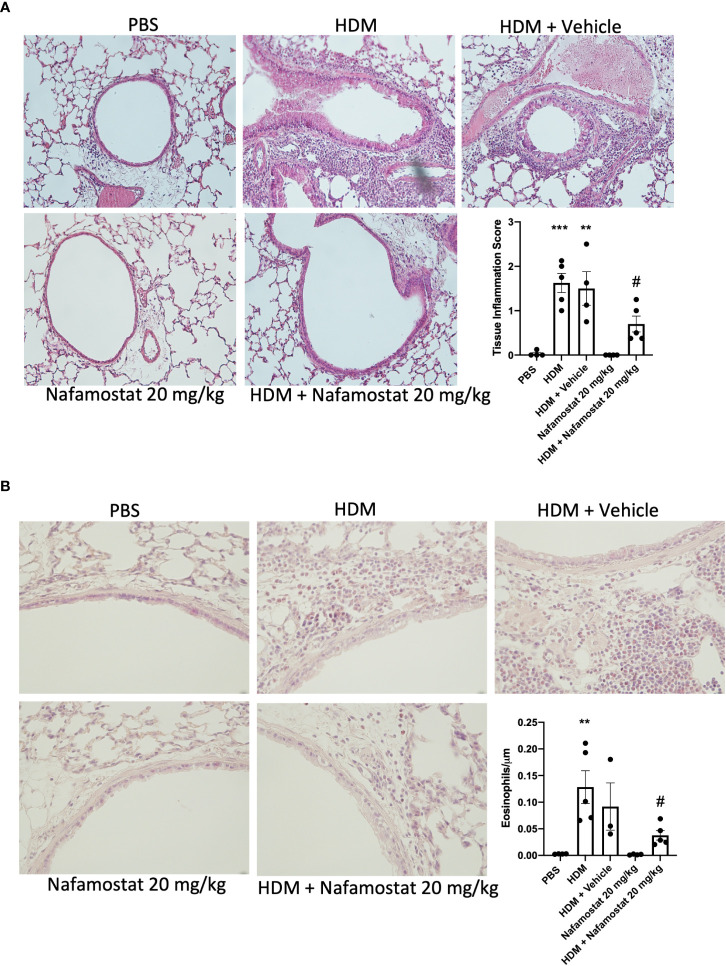
Effects of nafamostat on lung peribronchial inflammation and eosinophil infiltration in HDM-induced experimental asthma. Mice received either PBS or HDM extract twice a week for 6 weeks. Mice were treated with vehicle or with nafamostat (20 mg/kg) 30 min prior to each HDM instillation. Control mice were treated with PBS only. Peribronchial inflammation **(A)** and eosinophil infiltration around the airways **(B)** were assessed by hematoxylin and eosin (H&E) and chromotrope 2R staining, respectively. Representative images for H&E (x 20 original magnification) and chromotrope 2R staining (x 40 magnification) are shown. Data represent mean values ± SEM. **P < 0.01, ***P < 0.001 vs. the PBS group. ^#^ *P* < 0.05 vs. the HDM group. n = 3 – 5 mice per group. HDM, house dust mite.

To further evaluate the effect of nafamostat on lung inflammation, we examined effects on the peribronchial and perivascular infiltration of inflammatory cells by H&E staining, and infiltration of eosinophils into the lung tissue was assessed by chromotrope 2R staining. Consistent with the findings above, lung sections from HDM-sensitized mice showed a significant increase in peribronchial and perivascular inflammation (**Figure 2A**), which was mainly attributed to an excessive infiltration of eosinophils around the airways when compared to control mice (**Figure 2B**). Furthermore, treatment of the HDM-sensitized mice with nafamostat significantly attenuated the peribronchial and perivascular inflammation and caused a reduced infiltration of tissue eosinophils (**Figures 2A, B**). Together, these findings suggest that nafamostat could be beneficial to mitigate the hallmark features of allergic airway responses.

### Nafamostat mitigates HDM-induced inflammatory cytokine production

**Figure 3:**
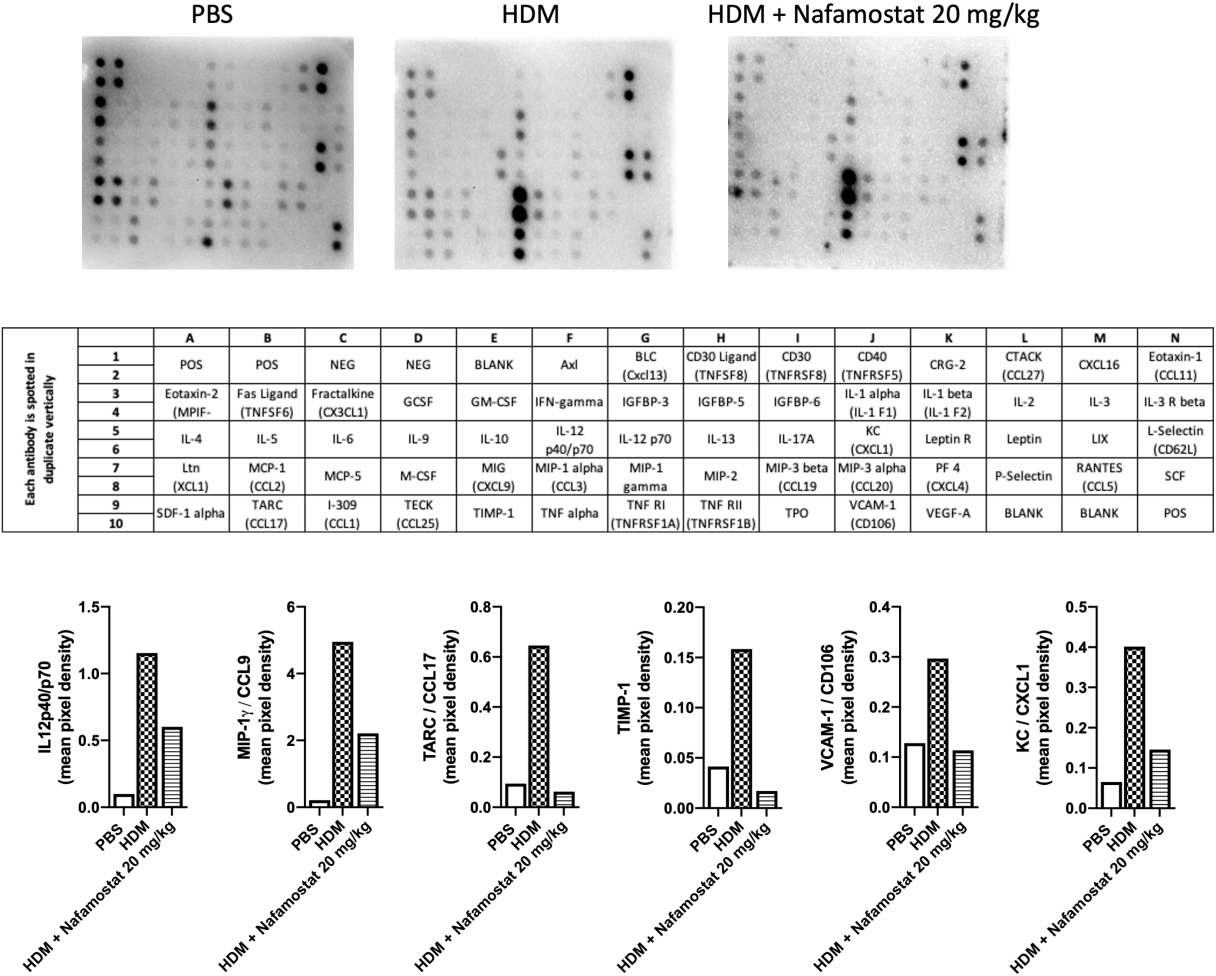
Effects of nafamostat on the release of cytokines into the BAL fluid of mice subjected to HDM-induced experimental asthma. Mice received either PBS or HDM extract twice a week for 6 weeks. Mice were treated with vehicle or with nafamostat (20 mg/kg) 30 min prior to each HDM instillation. Control mice were treated with PBS only. BAL fluid was collected, centrifuged and BAL fluid supernatants were pooled from each group and were then analysed with a RayBio mouse cytokine array. The pixel densities were semi-quantified using ImageJ protein array analyzer and are represented as mean pixel density. n = 4 mice per group. HDM, house dust mite.

Next, we examined the efficacy of nafamostat as an anti-inflammatory agent in allergic airway responses by measuring effects on inflammatory mediators recovered from the BAL fluid, by adopting a cytokine array approach. This assessment revealed increased levels of various cytokines/chemokines in BAL fluid from HDM-sensitized vs. control mice, including IL12p40/p70, MIP-1γ/CCL9, TARC/CCL17, TIMP-1, VCAM-1/CD106 and KC/CXCL1 (**Figure 3**). Notably, nafamostat treatment caused a profound reduction in the levels of each of these compounds in the BAL fluid, in most cases down to the baseline level (**Figure 3**). Of note, the HDM sensitization did not result in increased levels of Th2 cytokines (IL-4, IL-5, IL-13) in the BAL fluid.

### Nafamostat alleviates HDM-induced goblet cell hyperplasia and airway smooth muscle layer thickening

**Figure 4:**
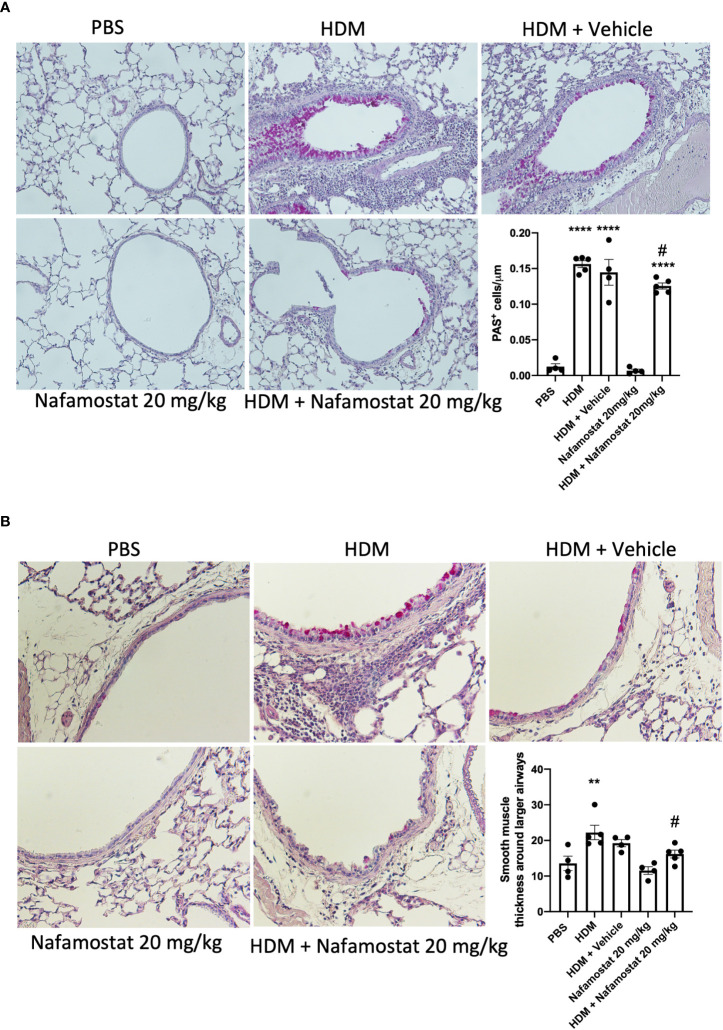
Effects of nafamostat on goblet cell hyperplasia and smooth muscle layer thickness in mice subjected to HDM-induced experimental asthma. Mice received either PBS or HDM extract twice a week for 6 weeks. Mice were treated with vehicle or with nafamostat (20 mg/kg) 30 min prior to each HDM extract instillation. Control mice were treated with PBS only. Goblet cell hyperplasia **(A)** and smooth muscle layer thickness around the primary bronchi **(B)** were quantified by Periodic Acid – Schiff (PAS) staining. Representative images for PAS staining (x 40 original magnification) are shown. Data represent mean values ± SEM. **P < 0.01, ****P < 0.0001 vs. the PBS group. ^#^ *P* < 0.05 vs. the HDM group. n = 4 – 5 mice per group. HDM, house dust mite.

Airway remodelling is a prominent feature of asthma, as characterized by increased smooth muscle cell proliferation and thickening of the smooth muscle layer, contributing to the airway narrowing and increased AHR. Considering our observed beneficial effects of nafamostat on HDM-induced airway inflammation and AHR, we next investigated the effect of nafamostat treatment on airway remodelling as manifested by goblet cell hyperplasia and smooth muscle layer thickening. Histological analysis demonstrated a significant rise in goblet cell density (assessed by PAS staining; **Figure 4A**) and an increased smooth muscle layer thickness (**Figure 4B**) in HDM-sensitized vs. control mice. Further, and consistent with its beneficial effect on other features of allergic airway inflammation, nafamostat treatment caused a modest, yet significant attenuation of the HDM-induced goblet cell hyperplasia and smooth muscle layer thickening (**Figures 4A, B**).

### Nafamostat treatment targets the expression of pro-inflammatory genes induced by HDM sensitization

**Figure 5:**
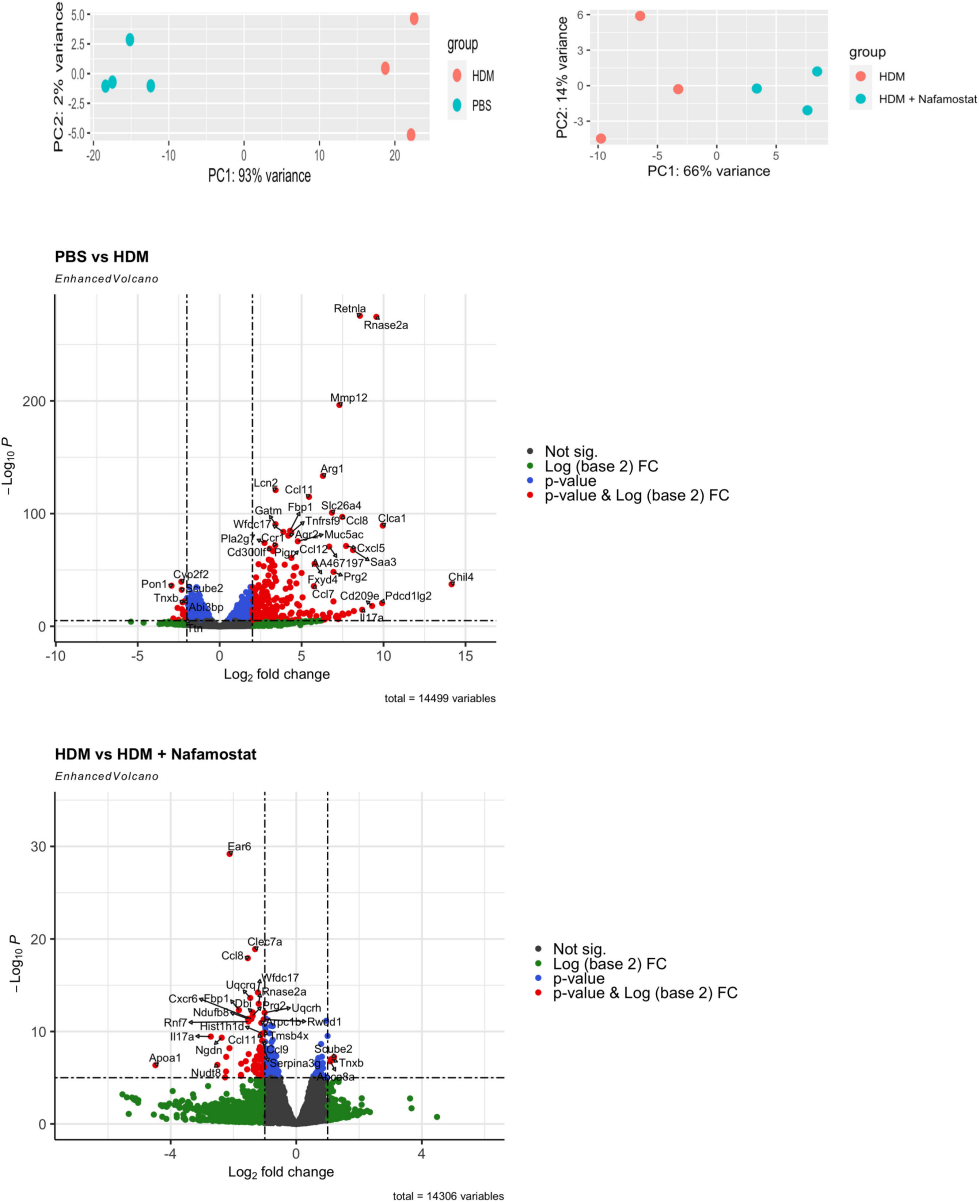
Effect of nafamostat on gene expression in lungs from mice subjected to HDM-induced experimental asthma. **(A, B)** Principal Component Analysis (PCA) of the comparison between the control- (PBS) vs. HDM **(A)** and the HDM vs. HDM + nafamostat (20 mg/kg) **(B)** groups. **(C, D)** Comparison of the differentially expressed genes (DEGs) between the control- (PBS) vs. HDM **(C)** and the HDM vs. HDM + nafamostat (20 mg/kg) **(D)** groups, by volcano plot analysis using the EnhancedVolcano package of R. Each dot represents a single gene. The x-axis depicts the log (base 2) of the fold change (FC), while the y-axis represents the negative log (base 10) of p-value. DEGs were identified as p < 0.05 and log2 FC ≥ 1.

To provide a more extended insight into the mechanism by which nafamostat ameliorates allergic airway inflammation, we next performed a transcriptomic analysis of how nafamostat affects the gene expression profiles in the lung, by adopting the Ampliseq platform. Transcripts with less than 30 counts were considered to be poorly expressed and were disregarded from the analysis. Visualization using principal component analysis (PCA) of the total lung transcriptome revealed a well-defined separation between control- (PBS) vs. HDM-sensitized mice (PC1 93% of variance between the groups and PC2 2% of variance) (**Figure 5A**) and also between HDM-sensitized vs. HDM-sensitized/nafamostat-treated mice (PC1 66% of variance between the groups and PC2 14% of variance) (**Figure 5B**). These findings suggest that the HDM-sensitization causes major effects on the lung transcriptome, and also that nafamostat has the capacity to modulate the expression of genes that are induced by HDM sensitization.

**Figure 6:**
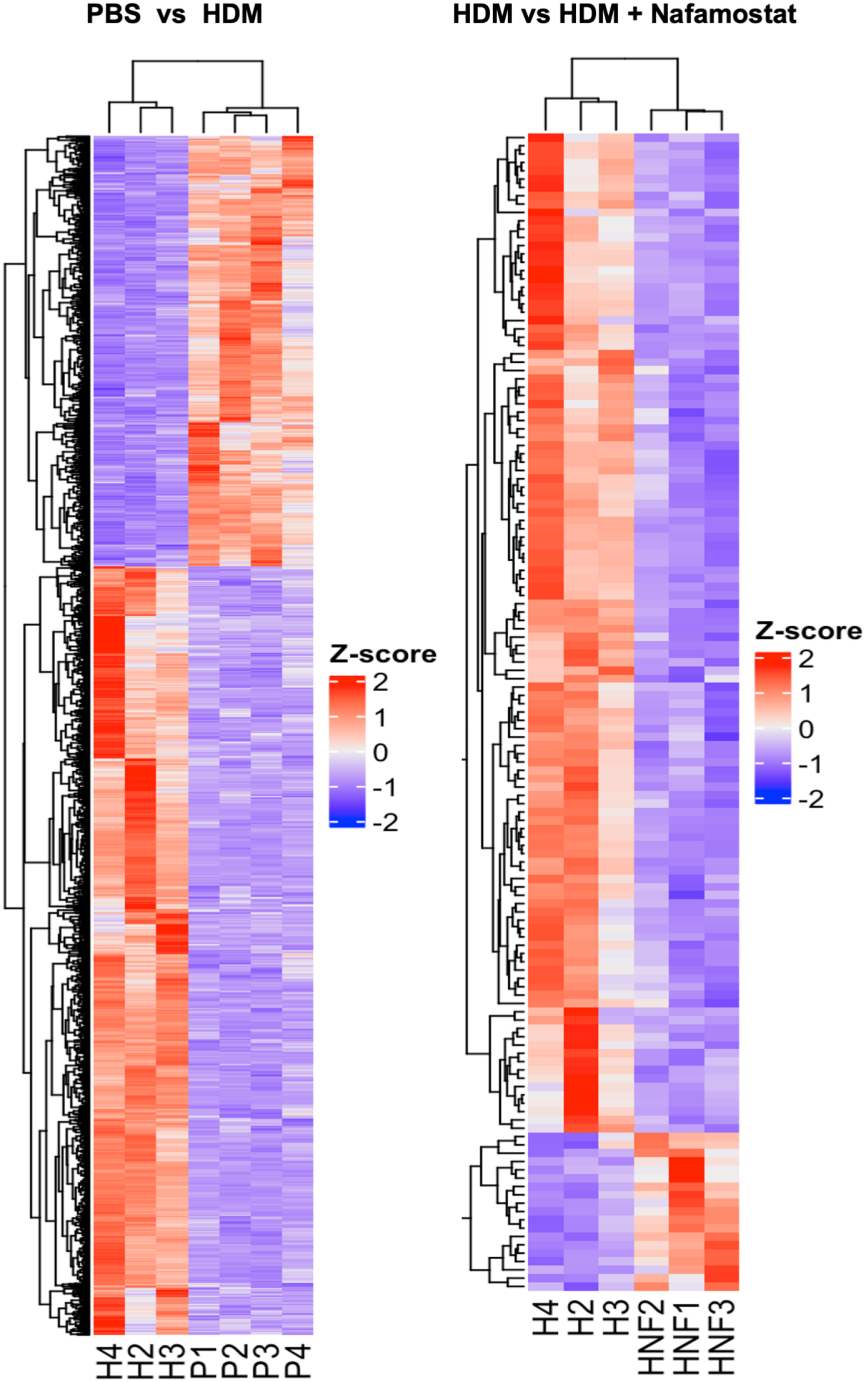
Heatmap constructed with hierarchical clustering to compare gene expression patterns in the control- (PBS) vs. HDM and the HDM vs. HDM + nafamostat (20 mg/kg) groups. n = 3 – 4 mice per group. HDM, house dust mite. n = 3 – 4 mice per group. HDM, house dust mite.

Differential gene expression analysis revealed that a total of 1680 out of 14499 transcripts were differentially (p<0.05; log2FC ≥ 1) expressed in HDM-sensitized vs. control mice, out of which 1077 genes were upregulated and 603 genes were downregulated in response to HDM sensitization (see **Supplementary Table 1**). Further, a comparison between HDM-sensitized vs. HDM-sensitized/nafamostat-treated mice revealed that 139 genes were differentially expressed, out of which 19 genes were upregulated and 120 genes were downregulated in response to nafamostat treatment (see **Supplementary Table 2**). Notably, the nafamostat treatment caused a reversal of the expression, back to baseline levels, of several of the genes that were induced by the HDM sensitization. The latter supports the notion that nafamostat, at least partly, can block the effects of HDM sensitization on global gene expression patterns in the lung. Using the enhanced volcano plot the profound effects of HDM sensitization on the lung transcriptome (**Figure 5C**; see also **Supplementary Table 1**), and the marked effects of nafamostat on the lung transcriptome in HDM-sensitized mice (**Figure 5D**; see also **Supplementary Table 2**) were visualized. The gene expression patterns in the lungs of control- vs. HDM-sensitized mice and in HDM-sensitized vs. HDM-sensitized/nafamostat-treated mice were also hierarchically clustered and presented as a heat map (**Figure 6**). Overall, these analyses reveal extensive effects of the HDM sensitization on the lung transcriptome, and also reveal profound effects of nafamostat treatment on the lung transcriptome after HDM sensitization.

**Figure 7:**
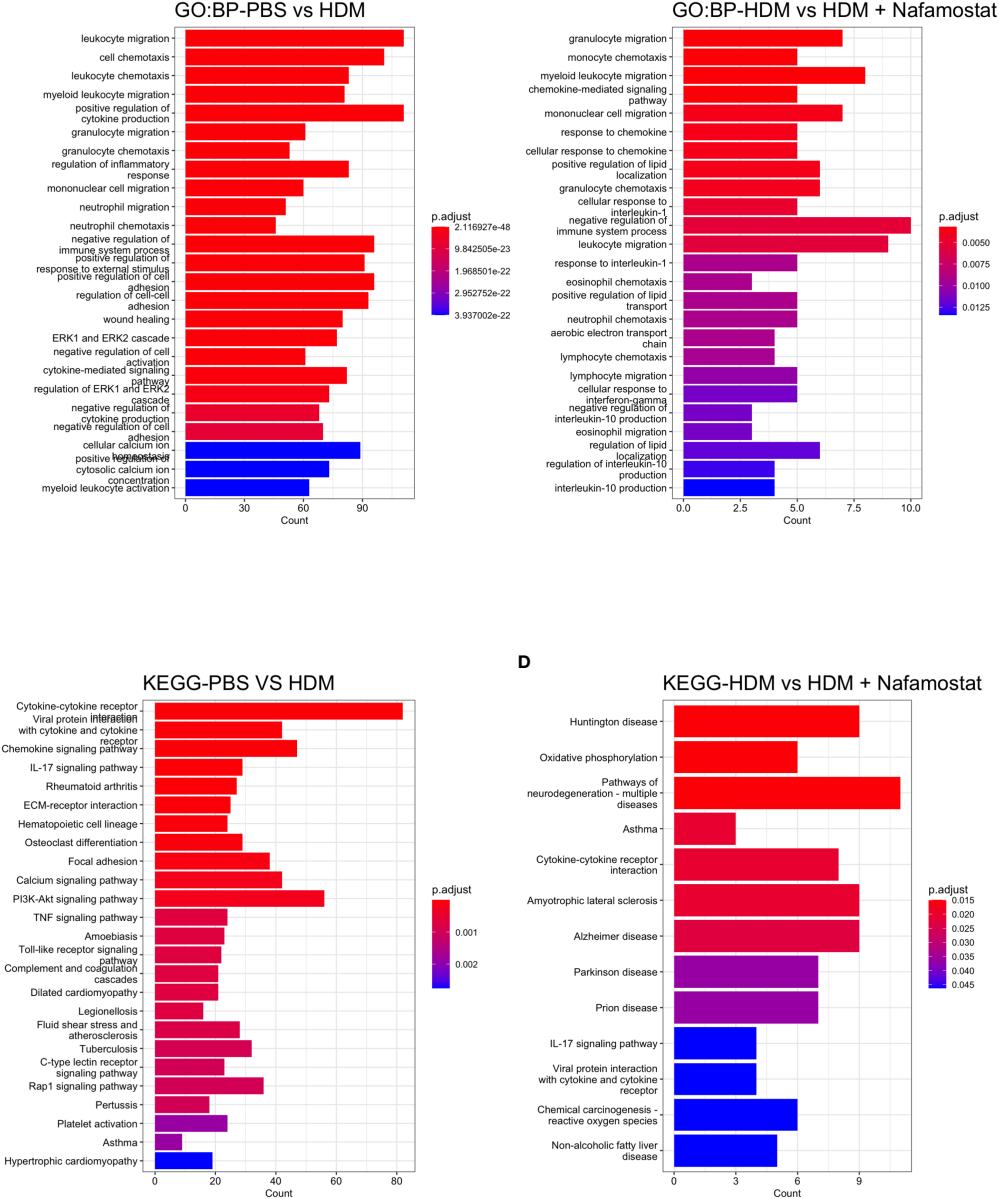
Overview gene enrichment analysis outlining the effect of nafamostat on HDM-induced features of asthma. The top 25 biological process (BP) **(A, B)** and the associated KEGG pathways **(C, D)** of the DEGs between the control- (PBS) vs. HDM and the HDM vs. HDM + nafamostat (20 mg/kg) groups were analysed using the enrichGO and enrichKEGG functions. The output of the analyses was visualized using bar plot function in clusterProfiler. For each plot, the x-axis represents the enriched gene counts and the y-axis represents a significant GO definition/KEGG pathway, respectively. n = 3 – 4 mice per group. HDM, house dust mite.

To further address the effects of nafamostat on parameters of HDM-induced allergic airway inflammation, enriched GO and KEGG analysis was performed (the top 25 categories were visualized). The GO analysis revealed that the HDM sensitization, as expected, caused significant effects on the transcription of genes associated with immune responses, including the categories: leukocyte chemotaxis and migration, regulation of cytokine production and cytokine mediated signalling (**Figure 7A**). Further, the GO analysis showed that several of these pathways were suppressed by nafamostat (**Figure 7B**), providing further support for an anti-inflammatory impact of nafamostat on allergic airway responses. Similarly, enriched KEGG analysis provided further support for a strong association between the HDM sensitization and pathways related to inflammation and immunity, including categories such as: cytokine-cytokine receptor interaction, chemokine signalling, IL-17 signalling pathway, toll-like receptor signalling, asthma, c-type lectin receptor pathway and ECM-receptor interaction (**Figure 7C**). Moreover, the KEGG analysis supported an anti-inflammatory impact of nafamostat on such pathways, including categories such as: cytokine-cytokine receptor interaction, asthma, and IL-17 signalling pathway (**Figure 7D**). Altogether, these findings highlight the profound effects of HDM sensitization on the lung transcriptome, and also that nafamostat has the capacity to partly block the effects of HDM sensitization on pro-inflammatory gene expression in the lungs.

**Figure 8:**
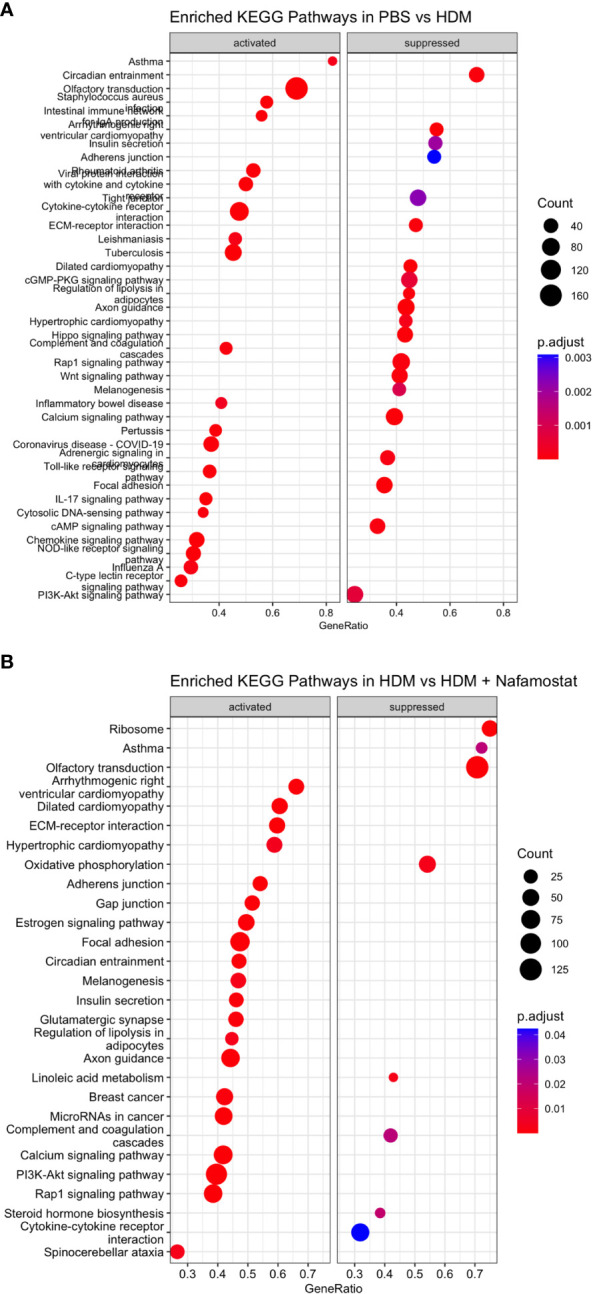
Gene set enrichment analysis revealed significantly enriched top 20 KEGG activated pathways (positive enrichment score) and supressed pathways (negative enrichment score) when comparing the control- (PBS) vs. HDM **(A)** and the HDM vs. HDM + nafamostat (20 mg/kg) **(B)** groups. The output of the analyses was visualized using dot plot function in clusterProfiler, with the y-axis representing the enriched KEGG pathway and the x-axis representing the gene ratio. The enrichment analysis was considered significant if p adjust ≤ 0.05. n = 3 – 4 mice per group. HDM, house dust mite.

Further analysis by gene set enrichment analysis on the KEGG pathways revealed that the HDM sensitization induced a positive enrichment of 62 pathways and negative enrichment of 49 pathways. The top positively enriched pathways (activated) that were mapped in response to HDM were: asthma, IL-17 signalling, linolenic acid metabolism, cytokine-cytokine receptor interaction, toll-like receptor signalling, chemokine signalling and c-type lectin receptor signalling (**Figure 8A** and **Supplementary Table 3**). The pathways that were negatively enriched (suppressed) in response to HDM sensitization included: Hippo signalling, Rap1 signalling, circadian entrainment, ECM-receptor interaction and Wnt signalling (**Figure 8A** and **Supplementary Table 3**). Moreover, a total of 46 pathways were positively enriched in HDM-sensitized/nafamostat-treated vs. HDM-sensitized mice, including several categories related to cardiomyopathy, and also including several of those categories that were associated with the HDM sensitization: Hippo signalling pathway, circadian entrainment, ECM-receptor interaction, Rap1 signalling (**Figure 8B** and **Supplementary Table 4**). Further, 8 pathways were shown to be suppressed by the nafamostat treatment. Importantly, “asthma” was one of the major pathways that was suppressed by the nafamostat treatment. Nafamostat also suppressed several other pathways, including the categories: linolenic acid metabolism, ribosome, oxidative phosphorylation, complement and coagulation cascades and cytokine-cytokine receptor interaction (**Figure 8B** and **Supplementary Table 4**). Hence, these findings provide further support for an ameliorating effect of nafamostat on the pro-inflammatory gene expression patterns that are associated with allergic airway inflammation.

**Figure 9:**
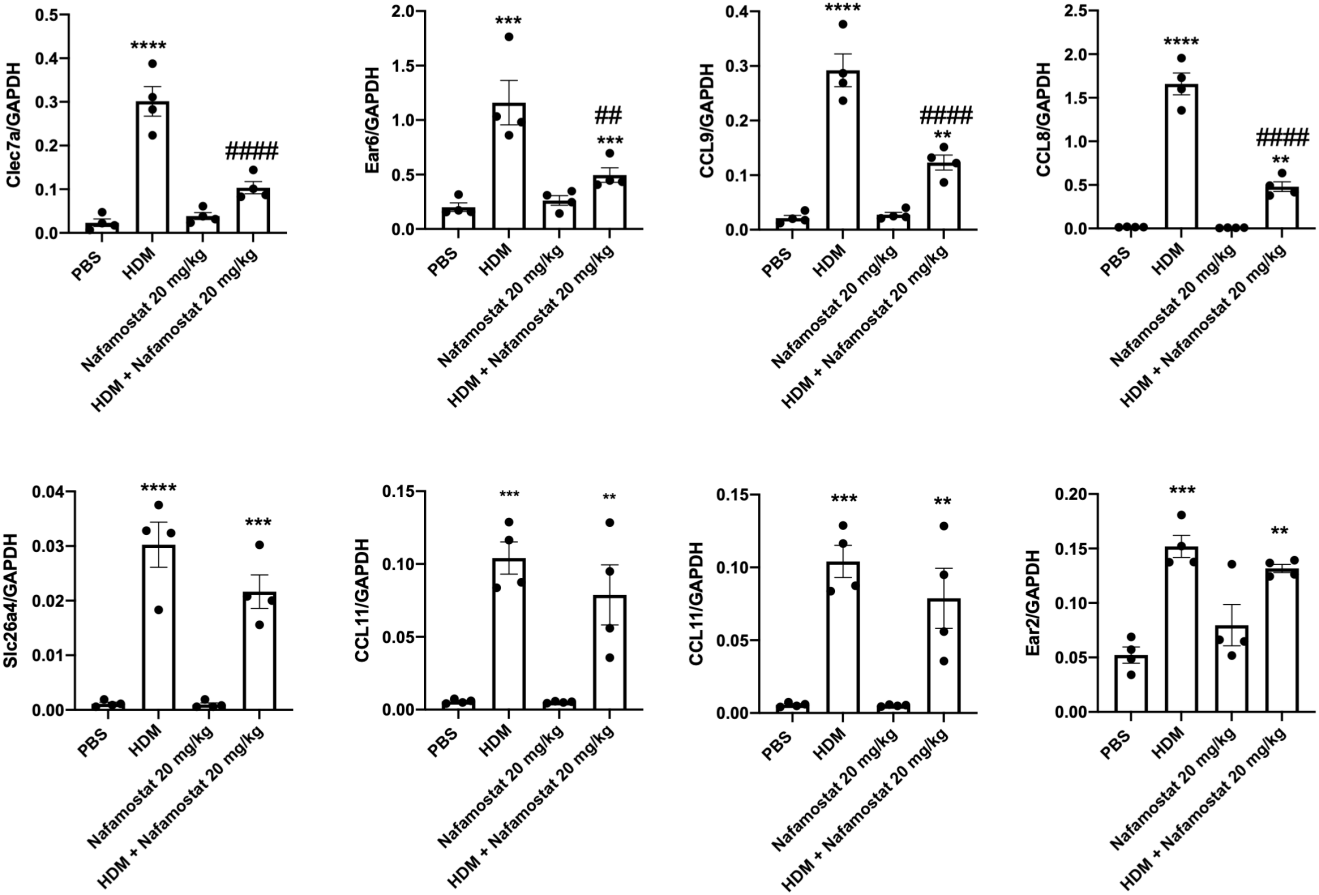
Nafamostat inhibits the expression of Dectin-1/Clec7a and chemokines in lungs of mice subjected to HDM-induced experimental asthma. Differential expression of genes in response to nafamostat was validated by qPCR analysis. The analysis included Dectin-1/Clec7a, Ear6, CCL9, CCL8, Slc26a4, CCL11, Fcer1g and Ear2. Data represent mean ± SEM. *p < 0.05, **p < 0.01, ***p < 0.001 and ****p < 0.0001 vs. the control (PBS) group. ^##^ *P* < 0.01, ^###^ *P* < 0.001 and ^####^ *P* < 0.0001 vs. the HDM group. n = 4 mice per group. HDM, house dust mite.

To further validate the effects of HDM sensitization and nafamostat on the gene expression patterns in the lung, we performed qPCR analysis. To this end, we focused on genes of particular relevance in asthmatic settings, including genes involved in eosinophil chemotaxis. These assessments thus included an analysis of the expression of Ear2, Ear6, Clec7a, Slc26a4, Fcer1g, CCL8, CCL9 and CCL11. In concordance with the transcriptome analysis, qPCR analysis supported that HDM-sensitized mice display a significant increase in the expression of all these genes in lung, when compared with control mice (**Figures 9**). Further, it was demonstrated that treatment of HDM-sensitized mice with nafamostat caused a significant attenuation of the expression of clec7a (Dectin-1), CCL8, CCL9 and Ear6 (**Figure 9**), and a trend of reduced Slc26a4 and CCL11 expression (**Figure 9**), whereas no effects of nafamostat on Fcerg1 or Ear2 expression were seen.

## Discussion

It has been extensively documented by using various experimental models of asthma that HDM exposure triggers airway inflammation associated with increased infiltration into the lungs of various immune cells, including eosinophils, lymphocytes, neutrophils and macrophages, and that this is accompanied by an increase in AHR, goblet cell hyperplasia, smooth muscle thickening and airway remodelling (30–32). In agreement with this, the current study supports that HDM sensitization promotes the development of such cardinal features of asthma. The present study also extends our knowledge of how HDM sensitization promotes the development of allergic airway inflammation, through an extensive transcriptome analysis of the gene expression alterations that occur in response to the allergic sensitization. Notably, the HDM sensitization caused profound alterations in the gene expression patterns of the lung, in particular by affecting pathway related to immunological processes.

Many of the inflammatory cells that are recruited by the HDM exposure have the capacity to secrete large amounts of proteases, and it is also notable that HDM extracts contain several types of proteases (8, 9). Hence, the HDM sensitization can potentially cause an imbalance between proteolytic activity and corresponding mechanisms to block such activities. Indeed, previous studies have suggested that such an imbalance between proteolytic activity and corresponding protease inhibition, executed by various physiological protease inhibitors such as α-1-antitrypsin, is one of the major factors in asthma pathogenesis (21). Hence, a potential strategy to intervene with asthma could be to administer inhibitors to those proteases that are operative in the pathogenesis of asthma, and several studies have previously reported beneficial effects of such inhibitors in asthmatic settings (15). Based on these notions, we here assessed the effects of nafamostat in HDM-induced airway inflammation and show that this protease inhibitor alleviates cardinal features of asthma, including the recruitment of airway inflammatory cells, peribronchial inflammation, AHR, goblet cell hyperplasia and smooth muscle layer thickening. Hence, our study supports the possibility of using nafamostat as a novel treatment regimen in human asthma.

Nafamostat is known as a highly selective inhibitor of mast cell tryptase (23), and our findings thus suggest that suppression of mast cell tryptase activity can account for the ameliorating effect of nafamostat on asthma features in the HDM model. In agreement with this scenario, mast cell tryptase has been extensively linked to the pathology of asthma, both in various animal models and also in severe asthma in humans [reviewed in (6)]. For example, administration of tryptase into lungs of sheep and mice are known to trigger asthma-like symptoms and exposure of human or guinea pig bronchi to tryptase leads to airway contraction (33–36). In further agreement with this, tryptase knockout mice develop less severe AHR than wild-type counterparts in an ovalbumin-based asthma model (37). However, it should be noted that nafamostat, in addition to inhibiting mast cell tryptase, can have effects on multiple other serine proteases, in particular at higher concentrations (23). Hence, we cannot exclude that the effects of nafamostat observed in this study is a result of the combined inhibition of mast cell tryptase and other types of serine proteases.

Regardless of the major protease target for nafamostat in the HDM-sensitization setting, our findings provide extensive insight into the mechanism behind its effects on allergic airway inflammation. One important finding was that nafamostat caused a reduction in the levels of several cytokines in the airway lumen of HDM-sensitized mice, including KC/CXCL1, TARC/CCL17 and MIP-1γ/CCL9. Of these, CXCL1 is involved in neutrophil recruitment (38), TARC/CCL17 promotes Th2 cell recruitment (32), and MIP-1γ/CCL9 has a role in monocyte recruitment and in the release of mediators from activated eosinophils (39–41). The suppression of these cytokines by nafamostat could thus, at least partly, account for its anti-inflammatory impact in the allergic setting. It is notable that several types of cytokines require proteolytic processing to become fully active (32, 42, 43). Hence, nafamostat-sensitive proteases could have a role in such activating proteolysis.

We also noted that nafamostat caused a reduction in the levels of VCAM-1, which has a key role in the recruitment of leukocytes in inflammatory settings (44, 45). The appearance of this compound in the BAL fluid of HDM-sensitized mice suggests that it is released by proteolytic shedding from epithelial cell surfaces, and our findings hence suggest that nafamostat could prevent such shedding by inhibiting proteases that are responsible for this.

An intriguing finding in this study was that HDM-associated airway inflammation is accompanied by an increased expression of Dectin-1/Clec7a, implying that signalling downstream of this receptor may contribute to the inflammatory response in this setting. In agreement with such a scenario, it was previously reported that HDM-induced airway inflammation was associated with increased populations of CD11b^+^ dendritic cells in mesenteric lymph nodes, and that this effect was blunted in Dectin-1-deficient (Clec7a^−/−^) mice (46). Notably, nafamostat caused a profound inhibition of the expression of Dectin-1/Clec7a, as judged both by the global transcriptomic analysis and by qPCR analysis, and it is thus conceivable that the anti-inflammatory effect of nafamostat may be related to its effects on Dectin-1/Clec7a expression. However, further studies are required to identify the exact mechanism by which nafamostat inhibits Dectin-1/Clec7a expression.

Further insight into the effect of nafamostat on HDM-associated airway inflammation came from an unbiased transcriptomics analysis. As expected, this revealed that the HDM sensitization caused extensive upregulation of a large number of genes coding for pro-inflammatory factors, including numerous genes related to leukocyte migration/chemotaxis, cytokine production and regulation of inflammatory responses. Importantly, as judged by pathway analysis, “asthma” represented the top upregulated gene category in HDM-sensitized vs. control animals, hence verifying that the adopted protocol produced an asthma-like condition. Hence, our findings provide an extensive insight into the effects of HDM sensitization on gene expression patterns in the lung. Considering that nafamostat ameliorates several hallmark features related to asthma (airway hyperresponsiveness, eosinophil recruitment, SMC thickness) it would be expected that genes related to such parameters were repressed by the inhibitor. Indeed, we noted that nafamostat caused suppression of corresponding gene categories, with one of the top repressed gene categories being “asthma”. Hence the transcriptomics analysis provides further support for a beneficial impact of nafamostat on allergic airway inflammation.

Overall, our findings are largely consistent with previous studies in which the effect of nafamostat has been assessed in various models of asthma (27–29). However, previous studies on this topic have only provided limited insight into the mechanism of action for nafamostat in such settings. Specifically, in the study by Ishizaki et al. (28), asthma-like conditions were induced by sensitization with ovalbumin, whereas the present study and the studies by Lin et al. and. Chen et al. (27, 29) utilized antigens from house dust mite, the latter representing a physiologically more relevant model for asthma than sensitization with ovalbumin. Further, whereas the effects of nafamostat on tissue inflammation and smooth muscle thickness was evaluated in the present study, these parameters were not assessed in the studies by Lin et al. and. Chen et al. (27, 29). Finally, this study, for the first time, evaluated global effects of nafamostat on gene expression patterns and cytokine output through unbiased approaches (transcriptomics, cytokine arrays) whereas the studies by Lin et al. and. Chen et al. focused on selected genes and cytokines (27, 29). Altogether, the present study has thus led to a deeper understanding of how nafamostat inhibition affects hallmark events associated with asthma, and has also provided extensive knowledge of how nafamostat affects the gene expression patterns during allergic airway inflammation induced by HDM sensitization. Notably though, further work will be required to identify the exact proteolytic target(s) for nafamostat-sensitive proteases in the allergic airway inflammation setting. We foresee that, based on this study, nafamostat may be further considered as a potential therapeutic agent in the treatment of human asthma.

## Materials and methods

### Animals

Female BALB/c mice (8 – 9 weeks of age) were purchased from Taconic Biosciences (Lille Skensved, Denmark). All procedures were performed at the Swedish University of Agricultural Sciences animal facility under protocols compliant with the EU Directive 2010/63/EU for animal experiments and approved by the local ethical committee (Uppsala djurförsöksetiska nämnd; Dnr 5.8.18-12873/2019). Mice were acclimatized for one week in the experimental room prior to the start of the experiment. Mice were lightly anaesthetized with isoflurane using a portable isoflurane vaporizer. Anaesthetized mice were instilled with 10 μg of house dust mite (HDM) extract reconstituted in 30 μl PBS (*Dermatophagoides pteronyssinus*, CiteQ BV, Groningen, Netherlands) intranasally twice a week for three weeks. Control mice received 30 μl PBS *via* the intranasal route. To investigate the efficacy of nafamostat on asthmatic features, nafamostat (20 mg/kg) (Sigma-Aldrich, St Louis, MO) or vehicle (3% acetic acid in PBS) were administered thirty minutes before each PBS and/or HDM instillation *via* the intraperitoneal route. A total of 48 mice were used in two individual experiments and the mice were allocated to the following experimental groups: PBS (n = 8), HDM (n = 9), HDM + vehicle (n = 9), nafamostat (20 mg/kg) (n = 8) and HDM + nafamostat (20 mg/kg) (n= 9).

### Measurement of airway hyperreactivity

Twenty-four hours after the last HDM instillation, airway hyperreactivity (AHR) was measured using a Buxco small ventilator (Buxco^®^ FinePointe Resistance and Compliance, Winchester, UK). Mice were anaesthetized with pentobarbital sodium (50 mg/kg; Sigma-Aldrich), and a tracheostomy was performed to insert a 20-gauge needle. Mice were kept under ventilation at 160 breaths/minute with a tidal volume of 0.25 mL. Mice were acclimatized to the ventilator before measuring the baseline. Lung resistance (R_L_) and dynamic compliance (C_dyn_) were measured using a dose-response curve for methacholine by administering increasing doses of nebulized methacholine (0 – 50 mg/mL; Sigma-Aldrich).

### Blood and bronchoalveolar lavage collection and analysis

Immediately after measuring lung function parameters, blood was collected and centrifuged at 2300 x g for 15 minutes at 4°C and serum was separated to measure HDM-specific IgE using ELISA (Chondrex, Woodinville, WA) following the manufacturer’s instructions. For bronchoalveolar lavage (BAL), the cannulated lungs were lavaged twice with 0.5 mL of sterile Hanks Balanced Salt Solution (HBSS). The collected BAL fluid was centrifuged at 600 x g for 10 min at 4°C. Supernatants were collected for analysis of cytokines and chemokines, while the cell pellet was resuspended in 1 mL sterile HBSS for enumeration of total and differential cell counts. For total cell counts, a hemocytometer was used. For differential leukocyte counts, cytospins were performed by spinning 100 µl cell suspensions onto glass slides at 26 x g for 5 min using a cytospin centrifuge. Cells were stained with May Grünwald/Giemsa, and a minimum of 200 cells per slide cells were counted. Cytokines levels in the BAL fluid were measured using the C3 Mouse Cytokine Array (RayBiotech, Norcross, GO) according to the manufacturer’s instructions by pooling the individual samples within the group. Signal pixel density assessment was conducted by using the ImageJ software (https://imagej.nih.gov/ij/).

### Lung histology

After BAL fluid collection, the right lung lobes were ligated, dissected and snap-frozen in liquid nitrogen and stored at -80°C for gene and protein analysis. The left lung lobes were inflated with 0.5 mL buffered formalin *via* the trachea cannula and trachea were sealed and fixed in buffered formalin. Formalin-fixed lobes were embedded in paraffin and 6 μm sections were prepared using a microtome. Sections were dewaxed and dehydrated by treatment with xylene, followed by a series of graded alcohol. Dewaxed lung sections were stained with haematoxylin and eosin (H&E) (30). Airway tissue inflammation was quantified by scoring the inflammatory cell infiltrate surrounding the airway using a semi-quantified score method ranging from 0-4 (0: no inflammatory cell infiltrates around the airway, 1: low-level cell infiltrates around part of the airway, 2: moderate cell infiltrates around part of or entire airway, 3: significant inflammatory cell infiltrate around part of or entire airway, 4: airway surrounded by inflammatory cell infiltrates). To study eosinophil infiltration, lung sections were stained with the chromotrope 2R (47). Bright red nuclei-stained cells around the airways were counted with a Nikon Microphot-FXA microscope using a 40 x objective lens and the Eclipse Net software.

To determine goblet cell hyperplasia and mucus production, airway sections were stained with periodic acid-Schiff’s (PAS). The number of goblet cells per μm of the airway epithelial layer was counted using a Nikon Microphot-FXA microscope with a 40 x objective lens and the Eclipse Net software (version 1.20, Developed by Laboratory Imaging, Prague, Czech Republic). The smooth muscle layer thickness around the airways was measured on the PAS-stained sections by measuring the thickness of every 30 μm basement membrane around the airways with a Nikon Microphot-FXA microscope and the Eclipse Net software; the average thickness was calculated. All quantitative histological assessments were performed and quantified by trained investigators in a blinded manner.

### Transcriptomic analysis

Snap-frozen upper portions of the left lung lobes from separate experiments were grounded in liquid nitrogen, and RNA was extracted using the Qiagen RNeasy^®^ plus mini kit (Hilden, Germany) following the manufacturer’s instructions. cDNA libraries were created and amplified using the Ion AmpliSeq™ Transcriptome Mouse Gene Expression Kit (Life Technologies, Carlsbad, CA) following the protocol of the manufacturer; the sequencing was performed on an Ion S5™ XL Sequencer (Thermo Fisher Scientific, Waltham, MA). Generated data had read lengths with an average of 98-110 bp and high mapping (95% of aligned bases). Normalized expression values generated by the Torrent Suite™ Software (version 5.10.1) were used for downstream differential gene expression analyses. The differential gene expression analysis was performed using the R package DESeq2 and genes with a log2FC ≥ 1 and an FDR-adjusted p-value <0.05 were considered as differentially expressed genes. To perform the gene enrichment analysis, we used the clusterProfiler package on the R program; the gene ontology with the biological process (BP) and KEGG pathway analyses were performed using the enrichGO and enrichKEGG functions in the clusterProfiler package (48). Functional enrichment analysis was performed using the Gene set enrichment analysis (GSEA) and the enrichment terms were considered significant with adjusted p < 0.05.

### Quantitative RT-PCR analysis

Quantitative RT-PCR (qPCR) analysis was used for further validation of the expression of selected genes. RNA concentration and purity was measured using a NanoDrop device (ND-1000; Nano Drop Technologies, Wilmington, Delaware). cDNA was prepared using 1 µg of total RNA and the iScript cDNA synthesis kit (Bio-Rad, Hercules, CA) following the manufacturer’s instructions. Validated primers for selected genes were purchased from BioRad (Hercules, CA) and qPCR analysis was performed on a C1000 Touch Thermal Cycler instrument (Bio-Rad, Hercules, CA) using the iTaq Universal SYBR Green Supermix (Bio-Rad). Each sample was run in duplicates and qPCR data analysis was performed using the Bio-Rad CFX Maestro program. Gene expression levels were normalized to glyceraldehyde 3-phosphate dehydrogenase (GAPDH) and the levels of gene expression were quantified using the 2^−ΔCT^ method

### Statistical analyses

Data are presented as mean values ± SEM, as analysed using GraphPad Prism 8.0. Two-way ANOVA statistical analysis was conducted to compare the *in vivo* lung function measurements between the experimental groups in response to the methacholine dose-response curve using the Bonferroni *post-hoc* analysis. For the rest of the experimental endpoints, one-way ANOVA was performed for statistical comparison between the treatment groups using Tukey *post-hoc* multi-comparison analysis. Differences between the experimental groups were regarded statistically significant when the p-value reached <0.05.

## Data availability statement

The datasets presented in this study can be found in online repositories. The names of the repository/repositories and accession number(s) can be found below: GSE223629 (GEO).

## Ethics statement

The animal study was reviewed and approved by Uppsala Djurförsöksetiska nämnd, Uppsala tingsrätt, Box 1113, 751 41 Uppsala, Sweden.

## Author contributions

VA designed and performed most of the experimental work, interpreted data and wrote the manuscript. IW contributed to the design of the study, and interpreted data. ST performed experimental work. SA contributed to the experimental work. SW contributed to the design of the study, interpreted data and contributed to the writing of the manuscript. GP conceived of the study, contributed to the design of the study, interpreted data and wrote the manuscript.All authors contributed to the article and approved the submitted version.

## References

[1] Holgate ST Davies DE Powell RM Howarth PH Haitchi HM Holloway JW. Local genetic and environmental factors in asthma disease pathogenesis: chronicity and persistence mechanisms. Eur Respir J (2007) 29(4):793. doi: 10.1183/09031936.00087506 17400878

[2] Kim HY DeKruyff RH Umetsu DT. The many paths to asthma: phenotype shaped by innate and adaptive immunity. Nat Immunol (2010) 11(7):577–84. doi: 10.1038/ni.1892 PMC3114595 20562844

[3] Allam V Chellappan DK Jha NK Shastri MD Gupta G Shukla SD. Treatment of chronic airway diseases using nutraceuticals: mechanistic insight. Crit Rev Food Sci Nutr (2022) 62(27):7576–90. doi: 10.1080/10408398.2021.1915744 33977840

[4] Allam V Paudel KR Gupta G Singh SK Vishwas S Gulati M. Nutraceuticals and mitochondrial oxidative stress: bridging the gap in the management of bronchial asthma. Environ Sci pollut Res Int (2022) 29(42):62733–54. doi: 10.1007/s11356-022-21454-w PMC9477936 35796922

[5] Cosmi L Liotta F Maggi E Romagnani S Annunziato F. Th17 cells: new players in asthma pathogenesis. Allergy (2011) 66(8):989–98. doi: 10.1111/j.1398-9995.2011.02576.x 21375540

[6] Pejler G. The emerging role of mast cell proteases in asthma. Eur Respir J (2019) 54(4):1900685. doi: 10.1183/13993003.00685-2019 31371445

[7] Ossovskaya VS Bunnett NW. Protease-activated receptors: contribution to physiology and disease. Physiol Rev (2004) 84(2):579–621. doi: 10.1152/physrev.00028.2003 15044683

[8] Randall TA London RE Fitzgerald MC Mueller GA. Proteases of dermatophagoides pteronyssinus. Int J Mol Sci (2017) 18(6):1204. doi: 10.3390/ijms18061204 28587273 PMC5486027

[9] Deb R Shakib F Reid K Clark H. Major house dust mite allergens dermatophagoides pteronyssinus 1 and dermatophagoides farinae 1 degrade and inactivate lung surfactant proteins a and d*. J Biol Chem (2007) 282(51):36808–19. doi: 10.1074/jbc.M702336200 17848554

[10] Georas SN Rezaee F. Epithelial barrier function: at the front line of asthma immunology and allergic airway inflammation. J Allergy Clin Immunol (2014) 134(3):509–20. doi: 10.1016/j.jaci.2014.05.049 PMC4170838 25085341

[11] Heijink IH Kuchibhotla VNS Roffel MP Maes T Knight DA Sayers I. Epithelial cell dysfunction, a major driver of asthma development. Allergy (2020) 75(8):1902–17. doi: 10.1111/all.14421 PMC7496351 32460363

[12] Trapani JA. Granzymes: a family of lymphocyte granule serine proteases. Genome Biol (2001) 2(12):REVIEWS3014. doi: 10.1186/gb-2001-2-12-reviews3014 11790262 PMC138995

[13] Menou A Duitman J Flajolet P Sallenave JM Mailleux AA Crestani B. Human airway trypsin-like protease, a serine protease involved in respiratory diseases. Am J Physiol Lung Cell Mol Physiol (2017) 312(5):L657–68. doi: 10.1152/ajplung.00509.2016 28235951

[14] Pham CT. Neutrophil serine proteases fine-tune the inflammatory response. Int J Biochem Cell Biol (2008) 40(6-7):1317–33. doi: 10.1016/j.biocel.2007.11.008 PMC2440796 18180196

[15] Guay C Laviolette M Tremblay GM. Targeting serine proteases in asthma. Curr Top Med Chem (2006) 6(4):393–402. doi: 10.2174/156802606776287054 16611150

[16] Fingleton B. Matrix metalloproteinases as regulators of inflammatory processes. Biochim Biophys Acta Mol Cell Res (2017) 1864(11 Pt A):2036–42. doi: 10.1016/j.bbamcr.2017.05.010 28502592

[17] Gandhi VD Shrestha Palikhe N Vliagoftis H. Protease-activated receptor-2: role in asthma pathogenesis and utility as a biomarker of disease severity. Front Med (Lausanne) (2022) 9:954990. doi: 10.3389/fmed.2022.954990 35966869 PMC9372307

[18] Takai T Kato T Hatanaka H Inui K Nakazawa T Ichikawa S. Modulation of allergenicity of major house dust mite allergens der f 1 and der p 1 by interaction with an endogenous ligand. J Immunol (2009) 183(12):7958–65. doi: 10.4049/jimmunol.0713276 19933866

[19] Kouzaki H Matsumoto K Kikuoka H Kato T Tojima I Shimizu S. Endogenous protease inhibitors in airway epithelial cells contribute to eosinophilic chronic rhinosinusitis. Am J Respir Crit Care Med (2017) 195(6):737–47. doi: 10.1164/rccm.201603-0529OC PMC5803669 27779422

[20] Biagini Myers JM Martin LJ Kovacic MB Mersha TB He H Pilipenko V. Epistasis between serine protease inhibitor kazal-type 5 (SPINK5) and thymic stromal lymphopoietin (TSLP) genes contributes to childhood asthma. J Allergy Clin Immunol (2014) 134(4):891–899.e3. doi: 10.1016/j.jaci.2014.03.037 24831437 PMC4186896

[21] Pfeffer PE Corrigan CJ. An imbalance between proteases and endogenous protease inhibitors in eosinophilic airway disease. Am J Respir Crit Care Med (2017) 195(6):707–8. doi: 10.1164/rccm.201610-2020ED 28294660

[22] Walsh DE Greene CM Carroll TP Taggart CC Gallagher PM O'Neill SJ. Interleukin-8 up-regulation by neutrophil elastase is mediated by MyD88/IRAK/TRAF-6 in human bronchial epithelium. J Biol Chem (2001) 276(38):35494–9. doi: 10.1074/jbc.M103543200 11461907

[23] Mori S Itoh Y Shinohata R Sendo T Oishi R Nishibori M. Nafamostat mesilate is an extremely potent inhibitor of human tryptase. J Pharmacol Sci (2003) 92(4):420–3. doi: 10.1254/jphs.92.420 12939527

[24] Park KT Kang DH Choi CW Cho M Park SB Kim HW. Is high-dose nafamostat mesilate effective for the prevention of post-ERCP pancreatitis, especially in high-risk patients? Pancreas (2011) 40(8):1215–9. doi: 10.1097/MPA.0b013e31822116d5 21775918

[25] Yates AG Weglinski CM Ying Y Dunstan IK Strekalova T Anthony DC. Nafamostat reduces systemic inflammation in TLR7-mediated virus-like illness. J Neuroinflamm (2022) 19(1):8. doi: 10.1186/s12974-021-02357-y PMC8734544 34991643

[26] Niemeyer BF Miller CM Ledesma-Feliciano C Morrison JH Jimenez-Valdes R Clifton C. Broad antiviral and anti-inflammatory efficacy of nafamostat against SARS-CoV-2 and seasonal coronaviruses in primary human bronchiolar epithelia. Nano Select (2022) 3(2):437–49. doi: 10.1002/nano.202100123 PMC8441815 34541574

[27] Chen C-L Wang S-D Zeng Z-Y Lin K-J Kao S-T Tani T. Serine protease inhibitors nafamostat mesilate and gabexate mesilate attenuate allergen-induced airway inflammation and eosinophilia in a murine model of asthma. J Allergy Clin Immunol (2006) 118(1):105–12. doi: 10.1016/j.jaci.2006.02.047 16815145

[28] Ishizaki M Tanaka H Kajiwara D Toyohara T Wakahara K Inagaki N. Nafamostat mesilate, a potent serine protease inhibitor, inhibits airway eosinophilic inflammation and airway epithelial remodeling in a murine model of allergic asthma. J Pharmacol Sci (2008) 108(3):355–63. doi: 10.1254/jphs.08162fp 19008643

[29] Lin CC Lin LJ Wang SD Chiang CJ Chao YP Lin J. The effect of serine protease inhibitors on airway inflammation in a chronic allergen-induced asthma mouse model. Mediators Inflammation (2014) 2014:879326. doi: 10.1155/2014/879326 PMC4142284 25180025

[30] Waern I Jonasson S Hjoberg J Bucht A Åbrink M Pejler G. Mouse mast cell protease 4 is the major chymase in murine airways and has a protective role in allergic airway inflammation. J Immunol (2009) 183(10):6369. doi: 10.4049/jimmunol.0900180 19841188

[31] Waern I Lundequist A Pejler G Wernersson S. Mast cell chymase modulates IL-33 levels and controls allergic sensitization in dust-mite induced airway inflammation. Mucosal Immunol (2013) 6(5):911–20. doi: 10.1038/mi.2012.129 23235745

[32] Malaviya R Zhou Z Raymond H Wertheimer J Jones B Bunting R. Repeated exposure of house dust mite induces progressive airway inflammation in mice: differential roles of CCL17 and IL-13. Pharmacol Res Perspect (2021) 9(3):e00770. doi: 10.1002/prp2.770 33929099 PMC8085917

[33] Molinari JF Scuri M Moore WR Clark J Tanaka R Abraham WM. Inhaled tryptase causes bronchoconstriction in sheep via histamine release. Am J Respir Crit Care Med (1996) 154(3 Pt 1):649–53. doi: 10.1164/ajrccm.154.3.8810600 8810600

[34] Barrios VE Middleton SC Kashem MA Havill AM Toombs CF Wright CD. Tryptase mediates hyperresponsiveness in isolated guinea pig bronchi. Life Sci (1998) 63(26):2295–303. doi: 10.1016/s0006-2952(03)00292-2 9877219

[35] Berger P Compton SJ Molimard M Walls AF N'Guyen C Marthan R. Mast cell tryptase as a mediator of hyperresponsiveness in human isolated bronchi. Clin Exp Allergy (1999) 29(6):804–12. doi: 10.1046/j.1365-2222.1999.00580.x 10336598

[36] Wong GW Foster PS Yasuda S Qi JC Mahalingam S Mellor EA. Biochemical and functional characterization of human transmembrane tryptase (TMT)/tryptase gamma. TMT is an exocytosed mast cell protease that induces airway hyperresponsiveness *in vivo via* an interleukin-13/interleukin-4 receptor alpha/signal transducer and activator of transcription (STAT) 6-dependent pathway. J Biol Chem (2002) 277(44):41906–15. doi: 10.1074/jbc.M205868200 12194977

[37] Cui Y Dahlin JS Feinstein R Bankova LG Xing W Shin K. Mouse mast cell protease-6 and MHC are involved in the development of experimental asthma. J Immunol (2014) 193(10):4783–9. doi: 10.4049/jimmunol.1302947 PMC4225174 25320274

[38] Rutledge H Aylor DL Carpenter DE Peck BC Chines P Ostrowski LE. Genetic regulation of Zfp30, CXCL1, and neutrophilic inflammation in murine lung. Genetics (2014) 198(2):735–45. doi: 10.1534/genetics.114.168138 PMC4196624 25114278

[39] Rose CE Jr. Lannigan JA Kim P Lee JJ Fu SM Sung SS. Murine lung eosinophil activation and chemokine production in allergic airway inflammation. Cell Mol Immunol (2010) 7(5):361–74. doi: 10.1038/cmi.2010.31 PMC3045045 20622891

[40] Le Floc’h A Allinne J Nagashima K Scott G Birchard D Asrat S. Dual blockade of IL-4 and IL-13 with dupilumab, an IL-4Rα antibody, is required to broadly inhibit type 2 inflammation. Allergy (2020) 75(5):1188–204. doi: 10.1111/all.14151 PMC7317958 31838750

[41] Branchett WJ Cook J Oliver RA Bruno N Walker SA Stölting H. Airway macrophage-intrinsic TGF-β1 regulates pulmonary immunity during early-life allergen exposure. J Allergy Clin Immunol (2021) 147(5):1892–906. doi: 10.1016/j.jaci.2021.01.026 PMC8098862 33571538

[42] Woo LN Guo WY Wang X Young A Salehi S Hin A. A 4-week model of house dust mite (HDM) induced allergic airways inflammation with airway remodeling. Sci Rep (2018) 8(1):6925. doi: 10.1038/s41598-018-24574-x 29720689 PMC5932037

[43] Berahovich RD Miao Z Wang Y Premack B Howard MC Schall TJ. Proteolytic activation of alternative CCR1 ligands in inflammation. J Immunol (2005) 174(11):7341. doi: 10.4049/jimmunol.174.11.7341 15905581

[44] Lee JH Sohn JH Ryu SY Hong CS Moon KD Park JW. A novel human anti-VCAM-1 monoclonal antibody ameliorates airway inflammation and remodelling. J Cell Mol Med (2013) 17(10):1271–81. doi: 10.1111/jcmm.12102 PMC4159019 23855490

[45] Fiscus LC Van Herpen J Steeber DA Tedder TF Tang ML. L-selectin is required for the development of airway hyperresponsiveness but not airway inflammation in a murine model of asthma. J Allergy Clin Immunol (2001) 107(6):1019–24. doi: 10.1067/mai.2001.114703 11398079

[46] Ito T Hirose K Norimoto A Tamachi T Yokota M Saku A. Dectin-1 plays an important role in house dust mite-induced allergic airway inflammation through the activation of CD11b+ dendritic cells. J Immunol (2017) 198(1):61–70. doi: 10.4049/jimmunol.1502393 27852745

[47] Lynch JP Werder RB Simpson J Loh Z Zhang V Haque A. Aeroallergen-induced IL-33 predisposes to respiratory virus–induced asthma by dampening antiviral immunity. J Allergy Clin Immunol (2016) 138(5):1326–37. doi: 10.1016/j.jaci.2016.02.039 27236500

[48] Wu T Hu E Xu S Chen M Guo P Dai Z. clusterProfiler 4.0: a universal enrichment tool for interpreting omics data. Innovation (2021) 2(3):100141. doi: 10.1016/j.xinn.2021.100141 34557778 PMC8454663

