# Supplementary material for: Nafamostat has anti-asthmatic effects associated with suppressed pro-inflammatory gene expression, eosinophil infiltration and airway hyperreactivity
Source: Front Immunol. 2023 Apr 21;14:1136780. doi: 10.3389/fimmu.2023.1136780 (PMC10160450; doi:10.3389/fimmu.2023.1136780)
Supplement: Supplementary file 1 [file DataSheet_1.pdf]

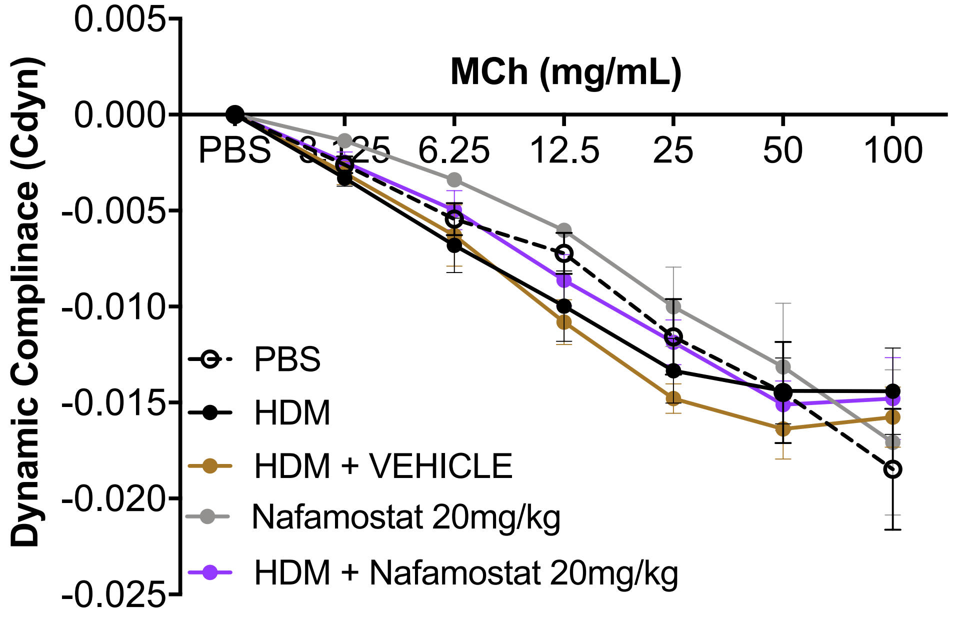

Supplementary Figure 1. House dust mite extract did not alter dynamic compliance (Cdyn). Mice received either PBS or HDM twice a week for 6 weeks. Mice were treated with vehicle or with Nafamostat (20 mg/kg) 30 min prior to each HDM instillation. Control mice were treated with PBS only. Dynamic Compliance (Cdyn) was measured using Buxco FinePointe series instrument. N = 4 – 5 mice per group. HDM = house dust mite.
